# Neuropsychiatric symptoms of dementia in those with and without a recorded history of psychological trauma: A comparative study from an Australian dementia support service

**DOI:** 10.1002/gps.6054

**Published:** 2024-01-07

**Authors:** Monica Cations, Mustafa Atee, Thomas Morris, Daniel Whiting

**Affiliations:** ^1^ College of Education, Psychology and Social Work Flinders University Adelaide South Australia Australia; ^2^ The Dementia Centre HammondCare Osborne Park Western Australia Australia; ^3^ Faculty of Health Sciences Curtin Medical School Curtin University Bentley Western Australia Australia; ^4^ Faculty of Medicine and Health Sydney Pharmacy School The University of Sydney Sydney New South Wales Australia; ^5^ Centre for Research in Aged Care School of Nursing and Midwifery Edith Cowan University Joondalup Western Australia Australia; ^6^ The Dementia Centre HammondCare St Leonards New South Wales Australia; ^7^ Faculty of Medicine and Health Sydney School of Public Health The University of Sydney Sydney New South Wales Australia

**Keywords:** dementia, neuropsychiatric symptoms, psychological trauma, traumatic stress

## Abstract

**Objective:**

To compare the number and severity of neuropsychiatric symptoms (NPS) and associated caregiver distress between those with and without a noted history of psychological trauma among those referred to a specialised national dementia NPS support service.

**Methods:**

This was a 5‐year retrospective observational study of records from the Dementia Support Australia NPS support service. NPS were reported by formal or informal caregivers at service entry using the Neuropsychiatric Inventory Nursing Home version or Questionnaire version. A history of psychological trauma was recorded in the person's social or medical history and/or endorsed as a contributor to NPS by a trained dementia consultant after a comprehensive clinical review. Regression was used to examine the impact of a recorded history of psychological trauma on NPS severity and associated caregiver distress, controlling for age and sex.

**Results:**

Among 41,876 eligible referrals with dementia, 6% (*n* = 2529) had some reference in their records to a history of psychological trauma. Referrals with a recorded history of psychological trauma were rated with a higher rate of both NPS severity (mean = 12.0) and associated caregiver distress (mean = 16.5) at service entry than those without a recorded history of psychological trauma (means = 10.7 and 14.5, respectively). A recorded history of psychological trauma was associated with higher odds of psychotic symptoms, agitation/aggression, irritability, disinhibition, affective symptoms and night‐time behaviours.

**Conclusions:**

Traumatic stress symptoms may represent a neglected target for intervention to reduce the impact of NPS in people with dementia.

## Key points

- Psychological trauma is a putative risk factor for dementia, and dementia onset can also trigger the re‐emergence or exacerbation of traumatic stress. However, little is known about how trauma contributes to neuropsychiatric symptoms (NPS) in dementia.
- People with dementia and a recorded history of psychological trauma were rated as having more frequent, more severe, and more distressing NPS than people without a recorded history of psychological trauma.
- Traumatic stress symptoms are modifiable and therefore may represent a neglected target for intervention to reduce the impact of NPS in people with dementia.

## 1 INTRODUCTION

Up to 70% of older people have experienced a psychologically traumatic event in their lifetime.^1^ Psychologically traumatic events are experienced or witnessed by an individual as physically or emotionally harmful or life threatening.^2^ While most people recover from traumatic stress, approximately 6%–8% of older survivors will meet criteria for post‐traumatic stress disorder (PTSD) at some time during their lifetime.^3^ Even without PTSD, experiencing psychological trauma can diminish coping and interpersonal skills such that older survivors report high rates of mental illness and social isolation.^4^

The interplay between psychological trauma and dementia is complex. While trauma is a putative risk factor for dementia, dementia onset can also trigger the re‐emergence or exacerbation of traumatic stress.^5^, ^6^ The mechanisms by which traumatic stress re‐emerges after dementia onset are not well understood, but are possibly related to progressive impairments in inhibitory control and other aspects of executive functioning that promote coping with and avoidance of traumatic memories.^7^, ^8^, ^9^

While trauma survivors may be more likely to develop dementia,^5^ very little research has examined how a trauma history affects experiences after dementia onset including the presence and severity of neuropsychiatric symptoms (NPS). NPS (e.g., aggression, agitation, sleep disturbance) occur in almost all people with dementia^10^ and are often highly disruptive and distressing experiences for the person, their family, and formal aged and acute care staff.^11^ Care staff consistently report lacking confidence and expertise to manage NPS.^12^

Psychological trauma may contribute to the presence and severity of NPS, and the link between the two is plausible for several reasons. First, psychological trauma can interrupt integration of sensory, emotional and cognitive information to the extent that seemingly neutral stimuli are perceived as threatening and trigger a distressing physiological response.^13^ Confusion and communication difficulties experienced by many people with dementia may contribute and trigger a biopsychosocial response that appears out of proportion (e.g., ‘explosive’ anger or fear, aggression, hoarding) or goes unnoticed (e.g., dissociation, hopelessness, withdrawal).^14^ Second, both psychological trauma and dementia can be characterised by impaired emotion regulation skills that are needed for coping with the threat response in safe ways.^15^ Third, dementia care services do not routinely identify trauma‐related needs and present high risk for re‐traumatisation where there are limited opportunities for a survivor to exercise choice and control.^16^

A recent observational study of 66 veterans with dementia identified that rejection of care was more commonly accompanied by aggression among those with a history of PTSD than those without PTSD.^17^ Similarly, a review of 30 case studies concluded that common NPS of dementia (e.g., anger and aggression, wandering) could be understood and treated as manifestations of traumatic stress symptoms (e.g., hypervigilance, difficulty regulating emotions, avoidance).^18^

However, how commonly psychological trauma contributes to NPS, in what ways, and the clinico‐demographic profile of this population have not yet been described. Limited data exist comparing NPS between people with and without a history of psychological trauma and how caregivers are affected by this. Large clinical services intended to support people with dementia experiencing NPS are well placed to fill this gap. Better characterisation and understanding of the relationship between psychological trauma and NPS will allow for the development and implementation of targeted treatment and support approaches.

This study aims to describe NPS in people with a recorded history of psychological trauma against those without such history who have been referred to Dementia Support Australia (DSA), a specialised national dementia support service that provides person‐centred non‐pharmacological strategies for NPS. Thus, the key objective of this study was to provide a comparative account of NPS characteristics and clinico‐demographics between the two groups over a period of 5 years, using DSA data. Our hypotheses were that (a) DSA referrals with a recorded history of exposure to traumatic events will have a more severe NPS profile (e.g., higher number and severity of individual NPS), and (b) overall caregiver distress would be higher among those caring for people with dementia with a recorded history of exposure to traumatic events.

## 2 MATERIALS AND METHODS

### 2.1 Ethical considerations

Ethical approvals for this study were granted by the University of New South Wales (HC190049), University of Sydney (2023/138), Edith Cowan University (2022‐03715) and Curtin University (HRE2023‐0069). The study was also approved (R258) by the Research Governance Office of HammondCare. All data were analysed in a retrospective and anonymous manner as per the granted waiver of informed consent of the ethics approval. The data were handled by a data custodian and in accordance with the Declaration of Helsinki of ethical principles, and the National Health and Medical Research Council's National Statement on Ethical Conduct in Human Research (2018). We used the Strengthening the Reporting of Observational Studies in Epidemiology Statement Checklist as a reporting guideline in our study.^19^

### 2.2 Study design, context, and reporting

This was a 5‐year retrospective observational (cross‐sectional) and comparative study examining the impact of a history of psychological trauma on the presence and severity of NPS and caregiver burden, for the period of 1 January 2018 and 31 December 2022. A similar methodology was previously described in Loi et al.^20^ The data were sourced from the DSA database.^21^ As part of service provision, DSA trained consultants and staff record demographic and clinical data (such as, age, dementia subtype) for people with NPS who were referred for behavioural support from two national programs, the Dementia Behaviour Management Advisory Service (DBMAS) for mild to moderate NPS and the Severe Behaviour Response Teams (SBRT) for moderate to severe NPS. These multimodal, person‐centred programs have been operational by DSA since 2016. Both programs are funded by the Australian Government to provide national support coverage for dementia related NPS across all states and territories in Australia.^21^

The programs are delivered by a large multidisciplinary team of dementia consultants, medical specialists (e.g., geriatricians, psychogeriatricians) and support staff. Dementia consultants comprise of health care professionals and allied health staff with accredited expertise and training in dementia, aged care, and behaviour support. Occupational backgrounds of consultants include (but are not limited to) nurses, occupational therapists, physiotherapists, social workers, psychologists, dieticians, speech pathologists and diversional therapists.^21^ To ensure consistent quality in the service provision, both DBMAS and SBRT programs deploy a case management framework. The framework encompasses onsite assessment with the referrals and caregivers to identify possible triggers of NPS, support strategies, interventions and recommendations, and ongoing visits and/or correspondence with the referrer to assess the impacts of the interventions.^21^

Eligibility criteria for DSA support include individuals with (1) a confirmed or probable diagnosis of dementia; (2) NPS related to the diagnosis of dementia; (3) NPS that impact their care and well‐being and/or their caregivers; and (4) consent received from the affected person with dementia or their responsible caregiver.^21^ Eligible individuals are referred to these programs via electronic means (website, email), fax, face‐to‐face while consultants are visiting a facility, or phone from multiple care settings, including residential aged care and community care. DSA programs are proven to be effective and feasible for reducing NPS and caregiver distress as demonstrated in a large Australian dementia sample (*n* = 5914).^21^

### 2.3 Participants

Participants were eligible for statistical analyses if were referred to DBMAS or SBRT programs during the 5‐year period of the study. When a referral was supported multiple times during the study period, only their earliest referral was used. Referrals were excluded from the analyses if any of the above criteria were not met. Analysis of NPS data included only participants for whom there was an available standardised assessment of NPS at service entry. An overview of participant inclusion is provided in Figure S1.

### 2.4 Study instruments

Demographic and clinical characteristics recorded at intake assessment and reported here include sex, age, dementia type, living situation, remoteness, and whether the person was recorded as a member of a special needs group (e.g., Lesbian, Gay, Bisexual, Trans, Intersex, Queer; veterans).

**Neuropsychiatric Symptoms**: In DSA programs, consultants routinely use the Neuropsychiatric Inventory (NPI) to assess formally NPS at service intake: the Nursing Home version (NPI‐NH)^22^ primarily for SBRT referrals and the Questionnaire version (NPI‐Q)^23^ mainly for DBMAS referrals. The NPI‐NH is completed by the consultant with a formal caregiver (i.e., aged care staff), whereas the NPI‐Q is completed by the consultant with an informal/family caregiver. Both NPI‐NH and NPI‐Q are psychometrically sound tools which evaluate 12 domains of different NPS such as agitation/aggression, depression and disinhibition.^22^, ^23^ Each domain is rated on the presence, severity (ranging from ‘mild’ 1 to ‘severe’3) and the distress or disruptiveness caused to caregivers (ranging from ‘not at all’ 0 to ‘very severe/extreme’5). The NPI‐NH also measures the frequency of each domain, ranging from ‘rarely’ (1) to ‘very often’ (4), and can be used to calculate the total number of endorsed domains (range: 0–12). Higher NPI scores indicate greater NPS severity, caregiver distress, and total endorsed domains associated with these symptoms. An NPI assessment is completed once for each referral at service intake.

**Psychological trauma**: The basic principle of the DSA model of care is the identification and modification of contributing factors (antecedents) to NPS. These factors are identified after DSA consultants conduct extensive onsite assessment with the person with dementia (i.e., the referral) and formal or informal caregivers of the person.^21^ This involves a comprehensive review of medical and social history. Under DSA programs, consultants audit medical records, conduct clinical observations and interviews, and conduct assessments with people (e.g., aged care staff, family members) who are familiar with the referral. Consultants can record that a person has a history of psychological trauma in their description of the person's social and medical history, and/or endorse a ‘tick box’ indicating psychological trauma as a contributing factor for NPS.

Using the DSA database, a two‐step process was employed to classify participants as having/not having had a traumatic experience. First, an initial set of target words were identified (e.g., ‘trauma’, ‘post‐traumatic stress’) and the descriptive social and medical histories of referrals were searched for these target words. After this, the initial hits were examined for false positives, and the target words were refined. A full list of search terms used for this analysis appear in Table S1. In addition to the key word searching, referrals where ‘Past Trauma’ was identified as contributing factor for NPS were classified into the trauma group. Where a person with dementia had multiple service records (i.e., due to having more than one care episode under the service), all records were reviewed for evidence of psychological trauma.

### 2.5 Statistical analyses

Demographic characteristics including sex, referral state, dementia subtype, living arrangement and remoteness were compared between the two groups using Pearson's chi‐square test. Cramer's *V* effect size was reported with 95% confidence interval (*CI*) alongside the *p* values for each comparison. Cramer's *V* effect sizes were interpreted in relation to the associated degrees of freedom of chi‐square test where medium effect size values are in the range of 0.13 (for 5 degrees of freedom) to 0.30 (for 1 degree of freedom).^24^, ^25^ The rates of the most frequent contributing factors in each group were compared using a two‐sample *Z‐test* of proportions with Yate's continuity correction and the 95% *CI* for the raw difference was calculated. The average total NPI severity, distress and number of domains were compared between groups using Welch's *t‐test*, with 95% *CIs* for the Cohen's *d* effect sizes. Descriptive statistics for the prevalence and average severity of each NPI domain for both groups were also calculated. A logistic regression model with severity scores for each domain predicting trauma status was used to examine the association between individual domains and a referral having a previous history of psychological trauma. Where domains were marked as absent, the domain severity score was included as 0. All statistical analyses were completed using R version 4.2.2.^26^

## 3 RESULTS

### 3.1 Demographic characteristics

The sample consisted of 41,876 referrals with dementia who were eligible for the analysis. Six per cent of the sample cases (*n* = 2529) had some reference in their records to psychological trauma (Table 1). In about half of these cases (*n* =1,254, 54.4%) the trauma history was noted in the person's social history. A trauma history was explicitly noted as a contributing factor for NPS in 836 cases (36.2%), and was noted in medical history (i.e., a diagnosis of PTSD) in 569 cases (24.7%). For 304 referrals (12.0%) a trauma history was noted in more than one location.

**TABLE 1:** Sample characteristics (*n* = 41,876).

	No trauma *n =* 39,347 (94.0%)	Trauma *n =* 2529 (6.0%)	Effect size (95% *CI*)
Age, mean (*SD*)	83.2 (8.6)	82.0 (8.8)	0.13 (0.09, 0.17), *p* < 0.001^a^
Female, *n* (%)	21,589 (54.9%)	1494 (59.1%)	0.02 (0.01, 0.03), *p* < 0.001^c^
Missing or indeterminate	283 (0.8%)	9 (0.4%)	
Dementia type, *n* (%)			
Alzheimer's disease	14,162 (36.0%)	891 (35.2%)	0.02 (0.00, 0.02), *p* = 0.013^c^
Dementia unspecified	10,726 (27.3%)	663 (26.2%)	
Vascular dementia	4767 (12.1%)	323 (12.8%)	
Mixed dementia	3232 (8.2%)	246 (9.7%)	
Other	1569 (4.0%)	119 (4.7%)	
Frontal lobe dementia	1165 (3.0%)	75 (3.0%)	
Lewy body dementia	1090 (2.8%)	84 (3.3%)	
Dementia in Parkinson's disease	529 (1.3%)	22 (0.9%)	
Missing	2107 (5.4%)	106 (4.2%)	
Location of referral, *n* (%)			
Residential aged care	33,776 (85.8%)	2295 (90.7%)	0.04 (0.02, 0.05), *p* < 0.001^c^
Referral/carer's home	4616 (11.7%)	170 (6.7%)	
Hospital/acute care	237 (0.6%)	18 (0.7%)	
Other	235 (0.6%)	20 (0.8%)	
Multi‐purpose service ^d^	192 (0.5%)	12 (0.5%)	
Respite care	59 (0.1%)	3 (0.1%)	
Indigenous flexible care service	19 (<0.1%)	3 (0.1%)	
Not stated or missing	213 (0.5%)	8 (0.3%)	
Remoteness, *n* (%)			
Major cities of Australia	27,597 (70.1%)	1705 (67.4%)	0.02 (0.00, 0.02), *p* = 0.006^c^
Inner regional Australia	7681 (19.5%)	574 (22.7%)	
Outer regional Australia	3175 (8.1%)	204 (8.1%)	
Remote Australia	341 (0.9%)	21 (0.8%)	
Very remote Australia	186 (0.5%)	12 (0.5%)	
Missing	367 (0.9%)	13 (0.5%)	
Service, *n* (%)			
Lower priority (DBMAS)	32,954 (83.8%)	2014 (79.6%)	0.05 (0.04, 0.06), *p* < 0.001^c^
Higher priority (SBRT)	4496 (11.4%)	457 (18.1%)	
Special needs group, *n* (%)			
Care leaver	36 (0.1%)	23 (0.9%)	0.82 (0.43, 1.21), *p* < 0.001^b^
Socially disadvantaged	96 (0.2%)	15 (0.6%)	0.35 (0.02, 0.67), *p* = 0.002^b^
Homeless	26 (0.1%)	6 (0.2%)	0.17 (−0.04, 0.38), *p* = 0.008^b^
Parents adoption	7 (<0.1%)	6 (0.2%)	0.22 (0.01, 0.43), *p* < 0.001^b^
Veteran	112 (0.3%)	57 (2.3%)	1.97 (1.37, 2.57), *p* < 0.001^b^
YOD (*n* = 41,629)	1273 (3.2%)	107 (4.2%)	0.99 (0.16, 1.82), *p* = 0.008^b^
LGBTIQ	110 (0.3%)	9 (0.4%)	0.08 (−0.18, 0.34), *p* = 0.613^b^
CALD (*n* = 34,850)	7938 (20.2%)	653 (25.8%)	4.75 (2.79, 6.71), *p* < 0.001^b^
Rural (*n* = 41,496)	11,383 (28.9%)	811 (32.1%)	3.03 (1.13, 4.93), *p* = 0.001^b^
First nations (*n* = 34,357)	498 (1.3%)	74 (2.9%)	1.75 (0.98, 2.53), *p* < 0.001^b^

Abbreviations: CALD, culturally and linguistically diverse; *CI*, Confidence interval; DBMAS, Dementia Behaviour Management Advisory Service; LGBTIQ, Lesbian, Gay, Bisexual, Trans, Intersex, Queer; *SD*, standard deviation; SBRT, Severe Behaviour Response Team; YOD, young onset dementia.

^a^ Cohen's *d*.

^b^ % Difference.

^c^ Cramer's *V* overall differences between groups.

^d^ Integrated health and aged care services delivered in regional and remote communities.

Referrals with dementia with a recorded history of psychological trauma were younger on average, more often female, more likely to live in a non‐metropolitan area, and more likely to reside in residential aged care than people without psychological trauma noted in their records. They were also more likely to be referred to the higher priority SBRT service, have a non‐Alzheimer dementia type (e.g., mixed dementia), and be referred to the service more than once (34.9%, compared to 23.7% in people without a noted history of psychological trauma). Referrals with a recorded history of psychological trauma were more often noted as belonging to a special needs group, including Care Leavers (i.e., people who as a child or youth spent time in institutional or other types of out‐of‐home care), people experiencing financial or social disadvantage (i.e., as defined by their need for welfare assistance), people experiencing or at risk for homelessness, people who were adopted, veterans, people with young onset dementia (i.e., with symptom onset before 65 years of age), people belonging to a culturally and linguistically diverse group, people living in a rural area, and First Nations people (i.e., Aboriginal and Torres Strait Islander Australians).

### 3.2 Neuropsychiatric symptoms (NPS)

An NPI assessment was completed for 1827 referrals with a recorded history of psychological trauma (72.2%), and 26,350 referrals without a recorded history of psychological trauma (67.0%). Among these, at intake to the service, referrals with dementia with a history of psychological trauma had higher NPS severity and caregiver distress scores than referrals without a recorded history of psychological trauma (Table 2). They also were recorded as exhibiting NPS in a slightly higher number of domains on average (mean = 5.2, standard deviation = 2.2) than those without a recorded history of psychological trauma (mean = 5.6, standard deviation = 2.2).

**TABLE 2:** Mean scores on Neuropsychiatric Inventory at service intake, by trauma history status.

	No trauma *n* =26,350 (94.0%)	Trauma *n* = 1827 (6.0%)	Cohen's *d* effect size (95% *CI*)
	Mean (*SD*)	Mean (*SD*)	
Total severity	10.7 (5.8)	12.0 (6.0)	0.21 (0.16, 0.26), *p* < 0.001
Total caregiver distress	14.5 (8.6)	16.5 (8.8)	0.23 (0.18, 0.27), *p* < 0.001
Total endorsed domains	5.2 (2.2)	5.6 (2.2)	0.19 (0.14, 0.23), *p* < 0.001
	Median 5, IQR 3	Median 5, IQR 3	

Abbreviations: *CI*, confidence interval; IQR, Interquartile range; *SD*, standard deviation.

**FIGURE 1:**
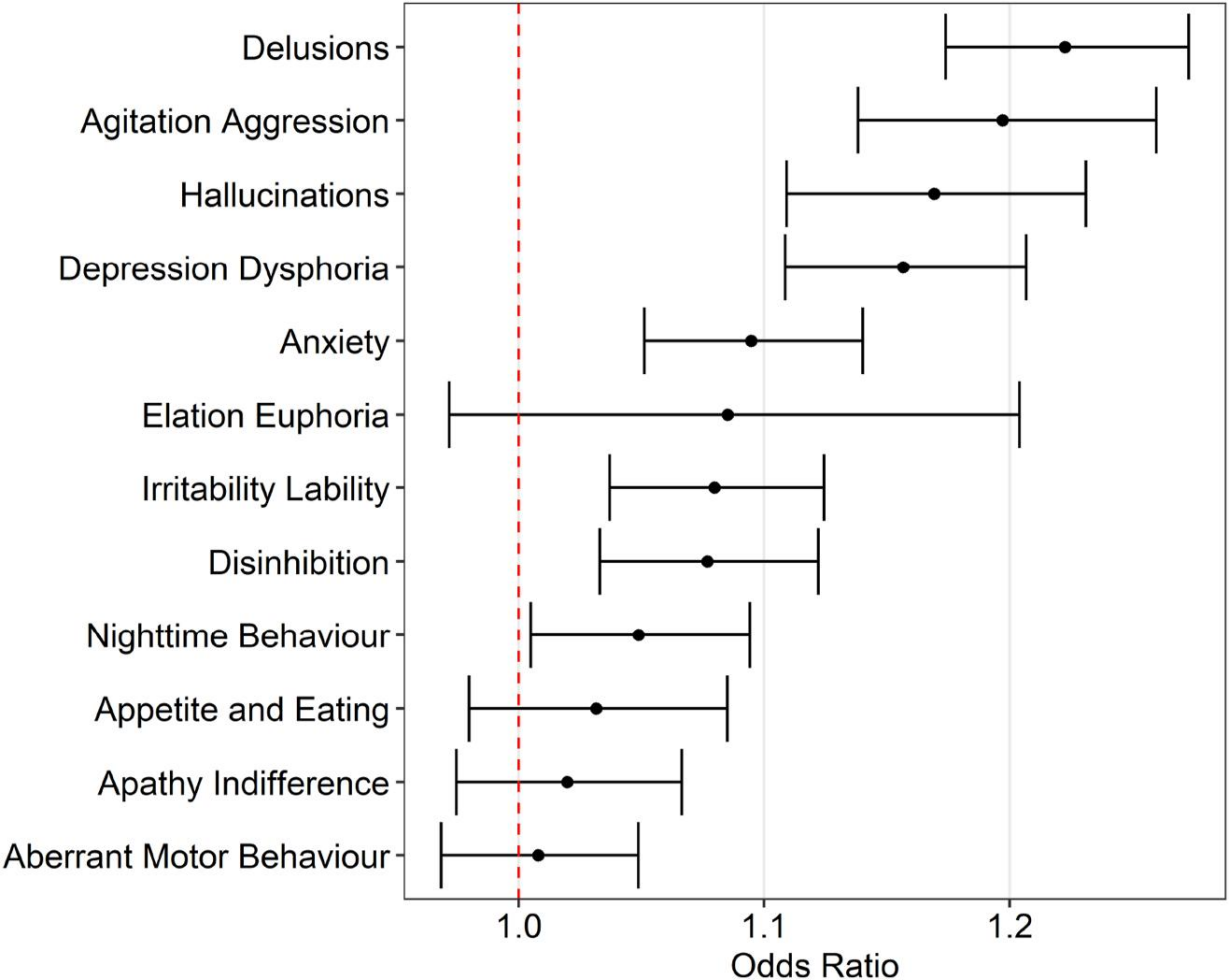
Results of logistic regression modelling assessing the association between a noted history of psychological trauma and presence of individual neuropsychiatric symptoms (measured by neuropsychiatric inventory) at service intake.

In linear regression modelling controlling for age and sex, a recorded history of psychological trauma was associated with a statistically significant higher rate of both NPS severity and associated caregiver distress at entry to the service (Table 3). In logistic regression modelling with individual NPS domains as the outcome variable, controlling for age and sex, a recorded history of psychological trauma was associated with higher odds of psychotic symptoms (delusions, hallucinations), agitation/aggression, irritability, disinhibition, affective symptoms (depression, anxiety) and night‐time behaviours (Figure 1). A recorded history of psychological trauma was not significantly associated with the presence of elation, appetite and eating change, apathy, or aberrant motor behaviour. Complete results of logistic regression modelling are presented in Table S2.

**TABLE 3:** Results of linear regression model (age‐ and sex‐adjusted) assessing association between a noted trauma history and neuropsychiatric symptom severity and caregiver distress on the Neuropsychiatric Inventory (NPI).

	Estimate (95% *CI*)	Standard error	*t*	*p* Value
NPI severity				
Intercept	12.60 (11.90, 13.29)	0.36	35.34	*p* < 0.001
Trauma history	1.18 (0.91, 1.46)	0.14	8.35	*p* < 0.001
Age	−0.02 (−0.03, −0.01)	0	−4.39	*p* < 0.001
Male sex	−0.79 (−0.93, −0.65)	0.07	−11.15	*p* < 0.001
NPI caregiver distress				
Intercept	17.30 (16.27, 18.33)	0.52	33	*p* < 0.001
Trauma history	1.87 (1.46, 2.28)	0.21	8.98	*p* < 0.001
Age	−0.03 (−0.04, −0.02)	0.01	−4.68	*p* < 0.001
Male sex	−0.81 (−1.01, −0.60)	0.1	−7.75	*p* < 0.001

Abbreviations: *CI*, confidence interval; NPI, Neuropsychiatric Inventory; *t*, *t*‐statistic for the regression coefficients.

## 4 DISCUSSION

This large multiyear study of people with dementia and NPS referred to a national specialist behaviour support service identified that those with a noted history of exposure to psychological trauma experience more severe and distressing NPS compared to those without a noted history of trauma exposure. As such, both hypotheses were supported by the results of this study. These results are consistent with a small existing body of evidence demonstrating a higher frequency of NPS among trauma survivors.^17^, ^18^ They suggest that psychological trauma may be an overlooked contributor to NPS and highlight the potential utility of interventions targeting traumatic stress in NPS management.

The field of research examining the relationship between psychological trauma and dementia is still emerging, with a small number of studies identifying that traumatic stress symptoms may re‐emerge after the onset of dementia in some people.^5^ Published case studies have suggested that common trauma responses to triggering stimuli can be mistaken for NPS in people with dementia.^27^ In this study, people with dementia and a noted history of psychological trauma were more likely to exhibit psychotic symptoms, agitation/aggression, irritability, disinhibition, affective symptoms and night‐time behaviours compared to those without a noted trauma history. The potential mechanisms of this relationship are not well understood. Some researchers have hypothesised that neurodegenerative disease may cause damage to factor systems responsible for fear activation, inhibiting the amygdala's inhibitory activity over traumatic memories and leaving people with dementia more susceptible to fear activation (and associated NPS) when confronted with reminders of traumatic events.^28^ People with dementia may also have less opportunity to avoid distressing stimuli, particularly where they are unable to leave their location or are receiving care that is not attuned to their needs.^18^

Identification and treatment of comorbid psychiatric conditions is recommended in the management of NPS in people with dementia.^29^ Recent work to develop and validate screening tools for traumatic stress symptoms in people with dementia may help clinicians to identify when trauma may be playing a role in the maintenance of NPS.^18^ Case studies have demonstrated that interventions to treat traumatic stress, including prolonged exposure and eye movement desensitisation and reprocessing, can be feasibly applied even with people with severe dementia.^30^ Whether these therapies can reduce the severity and impact of NPS is an important avenue for future research.

### 4.1 Strengths and limitations

The strengths of this study include the large sample size of people with dementia living both in the community and in residential aged care across all states and territories of Australia. The data were not collected for research purposes and therefore reflect the clinical practices of a large dementia behaviour support service embedded into the dementia care system in Australia.

Results should however be interpreted in the context of important methodological limitations. This analysis is observational and cross‐sectional in nature, and it is therefore not possible to infer causation in the relationships between variables. The recorded dementia diagnosis for referrals was noted by the DSA consultants based on existing and historic records, rather than consensus diagnostic criteria. Across the two groups, there were many referrals with an unspecified form of dementia. In addition, no data were available about the relative severity of the dementia, and this may have been a confounding factor in the relationship between a trauma history and NPS. In addition, an NPI assessment was not completed at intake for all participants, and referrals with a noted history of psychological trauma were more likely to receive an NPI at intake (72.2%) than referrals without a noted history of trauma (67.0%).

The identification of a history of psychological trauma was not systematic, instead noted by the DSA consultant based on clinical relevance and/or availability of information (e.g., socio‐medical history). Indeed, the prevalence of trauma history noted in the cohort (6%) is much lower than the estimated lifetime prevalence of exposure to traumatic events in older adults (approximately 70%^1^). We were also not able to examine how cognitive impairment impacted reporting of trauma history. Those identified with a trauma history within DSA records may therefore reflect a subset of trauma survivors for whom their traumatic experiences continue to affect their functioning, those who disclose their experiences, those where a trauma history was severe and well‐documented, and/or those with access to psychiatric care. No data were available about the nature of the trauma exposure, the lifetime or current post‐traumatic stress symptoms, comorbid psychiatric conditions, or history of treatment. As such, the differences identified between groups here may overstate the impact of trauma exposure on NPS for those who experienced less severe traumatic stress or who recovered from their traumatic stress.

Future population‐based studies of people with dementia will be important to establish the true nature and mechanisms of these relationships. Studies should consider multi‐methods approaches to gather details of the type of traumatic event exposure, timing of exposure, distress and symptomology, chronicity and severity of symptoms, and treatment outcomes. Administrative sources, family reports, and clinician reports will all be useful to supplement what a trauma survivor can or is willing to report themselves.

It is also possible that the care setting affected the relationship between NPS and trauma history. There are setting‐specific differences in the incidence, prevalence and persistence of NPS, and associated caregiving distress and burden. For instance, NPS are one of the primary contributors to residential aged care placement and are often more frequent and severe in residential aged care residents with dementia than those living in the community.^29^ Further, residential aged care residents are generally older, more frail, and presenting with a higher index of comorbidities and polypharmacy and more advanced stages of dementia.^31^ While most traumatic events are experienced prior to the diagnosis of dementia, manifestation of NPS and subsequent residential aged care placement,^1^ the identification of a trauma history in people with dementia is likely to largely rely on caregiver familiarity. It is possible that family caregivers of community‐dwelling people with dementia gain more familiarity with the life events of a person with dementia as a byproduct of their time spent providing care. On the other hand, formal assessments that occur in the process of residential aged care placement may promote identification. Future studies should examine the impact of participant living situation on the relationships identified here.

### 4.2 Implications and conclusions

This large study of data collected by a nationwide support service highlights the potential role of psychological trauma in the aetiology and maintenance of NPS in people with dementia. Previous studies have highlighted that the symptoms of PTSD and NPS can overlap, with shared mechanisms including hyperarousal and impaired emotion regulation skills.^18^ Importantly, traumatic stress symptoms are modifiable and therefore may represent a neglected target for intervention to reduce the impact of NPS in people with dementia.^17^

Even where treatment of traumatic stress symptoms is not available or feasible, amending care approaches to account for traumatic stress may be beneficial. Previous studies have argued that implementing trauma‐informed models of care into dementia care environments can improve the safety and predictability of care, reducing risk for re‐traumatisation and potentially exacerbating NPS.^16^, ^32^ Trauma‐informed care is a system‐level model of care that ensures that all care staff understand the potential impacts of traumatic events, can amend care to reduce the risk of re‐traumatisation (e.g. by preventing exposure to triggers), and can support the person to return to calm when dysregulated.^33^, ^34^ Thus, incorporating education and training for care staff in trauma‐informed care is an integral aspect of delivering better support to individuals with dementia. Published case series provide anecdotal evidence of successfully reducing the impact of NPS by amending care to account for traumatic stress symptoms, even without access to psychological or psychiatric expertise.^35^ Future studies assessing the implementation of trauma‐informed care models into dementia care environments will be helpful for assessing the potential benefits of these approaches.

## CONFLICT OF INTEREST STATEMENT

Nil to declare.

## ETHICS APPROVAL

Ethical approvals for this study were granted by the University of New South Wales (HC190049), University of Sydney (2023/138), Edith Cowan University (2022‐03715) and Curtin University (HRE2023‐0069). The study was also approved (R258) by the Research Governance Office of HammondCare.

## PATIENT CONSENT

Participants were people with dementia accessing the Dementia Support Australia support service, and consent was received from the affected person with dementia or their responsible caregiver before service engagement. Data collected during service provision were analysed in a retrospective and anonymous manner as per the granted waiver of informed consent of the ethics approval. The data were handled by a data custodian and in accordance with the Declaration of Helsinki of ethical principles, and the National Health and Medical Research Council's National Statement on Ethical Conduct in Human Research (2018).

## PERMISSION TO REPRODUCE MATERIAL FROM OTHER SOURCES

Not applicable.

## CLINICAL TRIAL REGISTRATION

Not applicable.

## Supporting information

Supporting Information S1

## Data Availability

All data used in this article will remain confidential to comply with the conditions of service provision of Dementia Support Australia (DSA).
