# Supplementary material for: Neuropsychiatric symptoms of dementia in those with and without a recorded history of psychological trauma: A comparative study from an Australian dementia support service
Source: Int J Geriatr Psychiatry. 2024 Jan 7;39(1):e6054. doi: 10.1002/gps.6054 (PMC10952246; doi:10.1002/gps.6054)
Supplement: Supplementary file 1 — Supporting Information S1 [file GPS-39-0-s001.docx]

# Supplementary Files

Supplementary Table S1. Search terms used to identify referrals with dementia with a noted history of psychological trauma.

| **Component** | **Breakdown** | **Interpretation** |
| --- | --- | --- |
| (?<!non\|not.{0,20}\|a\|poly\|neuro\|head \|minimal \|intracranial )trauma\\b |  | Hits for the word trauma (excluding the word trauma appearing as part of another word like traumatic), except where the word appeared following the specified words |
|  | trauma\\b | The target word not appearing as part of another word |
|  | (?<! ……. ) | The words appearing prior to the target word that would cancel out the hit |
|  | not.{0,20} | The word ‘not’ appearing anywhere from 0-20 characters before the word trauma (e.g. ‘did not experience any trauma’ |
| (?<!not \|not a )traumatic (?!brain\|head\|encephalopathy\|subarachnoid\|.+(haemorrhage\|hemorrhage\|fracture\|cortical)\|subdural\|pneumothorax\|intracerebral\|epilepsy\|intracranial \|olecranon bursitis) |  | The word traumatic not preceded by ‘not ‘ or ‘not a ‘ and not followed by the words specified in the brackets. |
|  | .+(….) | Any of the words specified in the brackets following the word traumatic at any point in the sentence. |
| posttraumatic stress\|post traumatic stress\|traumatic stress\|stress disorder\|ptsd |  | Any of the words appearing exactly as they are (i.e., no contextual modifications to whether they are a hit or not) |
| \\bpts |  | The acronym PTS |
|  | \\b | The marker for word boundary |

| Supplementary Table S2. *Results of logistic regression modelling assessing the association between a noted history of psychological trauma and presence of individual neuropsychiatric symptoms at service intake.* | | | | | |
| --- | --- | --- | --- | --- | --- |
| Term | Estimate | Odds Ratio | *SE* | *Z* | *p* Value |
| Aberrant Motor Behaviour | | | | | |
| (Intercept) | -1.12 | 0.33 [0.21, 0.51] | 0.23 | -4.82 | < .001 |
| Age | -0.02 | 0.98 [0.98, 0.99] | 0 | -6.47 | < .001 |
| Sex | -0.19 | 0.83 [0.75, 0.91] | 0.05 | -3.82 | < .001 |
| Severity | 0.01 | 1.01 [0.97, 1.05] | 0.02 | 0.39 | .697 |
| Agitation/Aggression | | | | | |
| (Intercept) | -1.49 | 0.23 [0.14, 0.36] | 0.24 | -6.27 | < .001 |
| Age | -0.02 | 0.98 [0.98, 0.99] | 0 | -6.37 | < .001 |
| Sex | -0.2 | 0.82 [0.74, 0.90] | 0.05 | -4.05 | < .001 |
| Severity | 0.18 | 1.20 [1.14, 1.26] | 0.03 | 6.96 | < .001 |
| Anxiety | | | | | |
| (Intercept) | -1.25 | 0.29 [0.18, 0.45] | 0.23 | -5.39 | < .001 |
| Age | -0.02 | 0.98 [0.98, 0.99] | 0 | -6.48 | < .001 |
| Sex | -0.15 | 0.86 [0.78, 0.95] | 0.05 | -3.04 | .002 |
| Severity | 0.09 | 1.09 [1.05, 1.14] | 0.02 | 4.37 | < .001 |
| Apathy/Indifference | | | | | |
| (Intercept) | -1.12 | 0.33 [0.21, 0.51] | 0.23 | -4.87 | < .001 |
| Age | -0.02 | 0.98 [0.98, 0.99] | 0 | -6.52 | < .001 |
| Sex | -0.19 | 0.83 [0.75, 0.91] | 0.05 | -3.84 | < .001 |
| Severity | 0.02 | 1.02 [0.97, 1.07] | 0.02 | 0.85 | .393 |
| Appetite and Eating | | | | | |
| (Intercept) | -1.13 | 0.32 [0.21, 0.51] | 0.23 | -4.9 | < .001 |
| Age | -0.02 | 0.98 [0.98, 0.99] | 0 | -6.5 | < .001 |
| Sex | -0.19 | 0.83 [0.75, 0.91] | 0.05 | -3.74 | < .001 |
| Severity | 0.03 | 1.03 [0.98, 1.08] | 0.03 | 1.2 | .231 |
| Delusions | | | | | |
| (Intercept) | -1.26 | 0.28 [0.18, 0.44] | 0.23 | -5.45 | < .001 |
| Age | -0.02 | 0.98 [0.98, 0.99] | 0 | -6.62 | < .001 |
| Sex | -0.16 | 0.86 [0.78, 0.94] | 0.05 | -3.13 | .002 |
| Severity | 0.2 | 1.22 [1.17, 1.27] | 0.02 | 9.73 | < .001 |
| Depression/Dysphoria | | | | | |
| (Intercept) | -1.3 | 0.27 [0.17, 0.43] | 0.23 | -5.59 | < .001 |
| Age | -0.02 | 0.98 [0.98, 0.99] | 0 | -6.47 | < .001 |
| Sex | -0.16 | 0.85 [0.77, 0.94] | 0.05 | -3.17 | .002 |
| Severity | 0.15 | 1.16 [1.11, 1.21] | 0.02 | 6.73 | < .001 |
| Disinhibition | | | | | |
| (Intercept) | -1.19 | 0.30 [0.19, 0.48] | 0.23 | -5.14 | < .001 |
| Age | -0.02 | 0.98 [0.98, 0.99] | 0 | -6.41 | < .001 |
| Sex | -0.2 | 0.82 [0.74, 0.90] | 0.05 | -3.97 | < .001 |
| Severity | 0.07 | 1.08 [1.03, 1.12] | 0.02 | 3.52 | < .001 |
| Elation/Euphoria | | | | | |
| (Intercept) | -1.14 | 0.32 [0.20, 0.50] | 0.23 | -4.93 | < .001 |
| Age | -0.02 | 0.98 [0.98, 0.99] | 0 | -6.42 | < .001 |
| Sex | -0.19 | 0.83 [0.75, 0.91] | 0.05 | -3.76 | < .001 |
| Severity | 0.08 | 1.09 [0.97, 1.20] | 0.05 | 1.5 | .134 |
| Hallucinations | | | | | |
| (Intercept) | -1.23 | 0.29 [0.19, 0.46] | 0.23 | -5.31 | < .001 |
| Age | -0.02 | 0.98 [0.98, 0.99] | 0 | -6.27 | < .001 |
| Sex | -0.18 | 0.83 [0.76, 0.92] | 0.05 | -3.65 | < .001 |
| Severity | 0.16 | 1.17 [1.11, 1.23] | 0.03 | 5.88 | < .001 |
| Irritability/Lability | | | | | |
| (Intercept) | -1.21 | 0.30 [0.19, 0.47] | 0.23 | -5.21 | < .001 |
| Age | -0.02 | 0.98 [0.98, 0.99] | 0 | -6.54 | < .001 |
| Sex | -0.2 | 0.82 [0.75, 0.91] | 0.05 | -3.92 | < .001 |
| Severity | 0.08 | 1.08 [1.04, 1.12] | 0.02 | 3.73 | < .001 |
| Night-time Behaviour | | | | | |
| (Intercept) | -1.13 | 0.32 [0.21, 0.51] | 0.23 | -4.92 | < .001 |
| Age | -0.02 | 0.98 [0.98, 0.99] | 0 | -6.6 | < .001 |
| Sex | -0.19 | 0.82 [0.75, 0.91] | 0.05 | -3.9 | < .001 |
| Severity | 0.05 | 1.05 [1.00, 1.09] | 0.02 | 2.2 | .028 |

Abbreviations: *SE*, Standard error; *Z*, Standard score

Referred to DBMAS or SBRT programs during the 5-year period of the study and completed intake assessment

*n* = 41,876

Recorded history of psychological trauma

*n* = 2,529 (6.0%)

No recorded history of psychological trauma

*n* = 39,347 (94.0%)

No recorded history of psychological trauma +

NPI assessment completed at service intake

*n* = 26,350 (62.9%)

Recorded history of psychological trauma + NPI assessment completed at service intake

*n* = 1,827 (4.4%)

Figure S1. Participant inclusion flow chart.
